# Assessment of Female Hormonal Influence on COVID-19 Vaccine Response: A Prospective Cohort Study

**DOI:** 10.7759/cureus.54417

**Published:** 2024-02-18

**Authors:** Suganya Panneer Selvam, Ramya Ramadoss, RajeshKumar Shanmugam, Sandhya Sundar, Lakshmi TA, Pratibha Ramani

**Affiliations:** 1 Oral Pathology and Oral Biology, Saveetha Dental College and Hospitals, Saveetha Institute of Medical and Technical Sciences, Chennai, IND; 2 Pharmacology, Saveetha Dental College and Hospitals, Saveetha Institute of Medical and Technical Sciences, Chennai, IND; 3 Oral Pathology, Saveetha Institute of Medical and Technical Sciences, Chennai, IND

**Keywords:** menstruation, menopause, cortisol, igg, vaccines, saliva, covid

## Abstract

Introduction: The diversity of oral epithelial cells offers potential viral infection sites. The lower level of ACE2 inhibitors in women's blood renders them more resistant to coronavirus disease 2019 (COVID-19). In order to determine the effect of COVID-19 vaccination on female hormones, salivary levels of total antibody, immunoglobulin G (IgG), and cortisol were measured in young and elderly women.

Methods: Saliva samples from 88 participants were collected and subjected to ELISA for detecting total antibody, IgG, and cortisol.

Results: Women who were infected with COVID-19 and who completed two doses of vaccination had more IgG antibodies when compared to the uninfected individuals/single-dose/non-vaccinated individuals. The cortisol levels in post-menopausal women were higher than those in women with normal menstrual cycles, and the difference was statistically significant (P-value 0.00). The increased cortisol levels were well correlated with increased levels of IgG antibodies which was statistically significant (Spearman rho P value 0.00)

Conclusions: COVID variants will continue to mutate and evolve as long as the epidemic persists. The higher cortisol and IgG antibodies produced by female hormones protect them from COVID-19 infection.

## Introduction

Coronavirus disease 2019 (COVID-19) is the fifth-largest pandemic in recent history, with the earliest commencement of symptoms occurring in the first week of December 2019 in Wuhan, China. It is the deadliest pandemic to date because it spreads through infectious particles expelled during coughing and sneezing. Hence, salivary secretions will continue to be the primary source of infection [1]. Currently, strains Alpha (B.1.1.7), Beta (B1.351), Gamma (P.1), and Delta (B1.617.2) are of concern, and the virus keeps evolving and disseminating. The Omicron variant is a novel variety with remarkable transmissibility [2]. Evidence from previous studies found that the concentration of ACE2 in the blood of men is higher than that of females, making them more susceptible to COVID-19 infection. This strongly recommends gender-based investigation and treatment [3].

Vaccine development is an expensive and time-consuming process, and it takes years to produce a licensed vaccine. In order to produce vaccines during a pandemic without knowing the exact efficacy of the vaccines, substantial production facilities and significant financial resources are required [4,5]. During the pandemic, the most extensively distributed vaccines in India were by Serum Institute of India (COVISHIELD) and Bharat Biotech (COVAXIN) [6,7]. The Long COVID syndrome is an outcome of the COVID-19 vaccine's detrimental impacts on multiple systems, including the endocrine system. Fewer studies have been conducted on the topic, so the effect on the reproductive system is still unclear.

Due to the variety of oral epithelial cells, there are numerous attachment sites for viral infection. IgG antibodies are detectable in saliva for at least nine months during COVID-19 infection, whereas IgA antibodies develop rapidly within one week of infection onset and diminish after four to six weeks [8]. The specificity of saliva samples for identifying asymptomatic carriers is high, and sensitivity increases with the infection stage [9]. Saliva remains the simplest technique for evaluating the mucosal and systemic immune reactions to any infection or vaccination. Consequently, we detected anti-COVID-19 antibodies in the saliva of women with normal menstrual cycles and menopause.

The body's response to physiological stress, which affects immunological control, results in an increase in serum cortisol levels [10]. Patients with the COVID-19 infection or after treatment for the same exhibit elevated stress levels, indicating that COVID-19 patients have a greater acute cortisol stress response. Women's cortisol levels rise during the menopause transition and after menopause [11]. Stress levels and the menstrual cycle of women are intrinsically linked. Consequently, it is considered a physiological indicator of chronic stress. It is challenging to detect free cortisol in females because it is bound to globulin and cannot be measured in a laboratory. In contrast, salivary cortisol can overcome this barrier, as only free cortisol can do so. In the presence of estrogen, the liver produces cortisol-binding globulin, resulting in a deficiency of free cortisol [12]. Both quantitative and qualitative measurements of free cortisol can be demonstrated using cortisol extracted from saliva. In this study, the stress levels of females following COVID-19 vaccination are evaluated. 

This article measures the levels of IgG antibodies, total antibodies, and cortisol in the unstimulated saliva of women with normal menstrual cycles and menopausal women in order to emphasize the influence of SARS-CoV and the effects of COVID-19 vaccination on the female reproductive system. 

## Materials and methods

Ethical approval for the study

The Saveetha Institute of Medical and Technical Sciences Institutional Ethical Committee in Chennai granted ethical approval for the investigation (IHEC/SDC/FACULTY/21/OPATH/196). Informed consent was obtained from all participants.

Sample size

The study involved the random selection of 88 female participants from the outpatient population. Clinical data were gathered from these participants, including age, medication history, history of current illness, history of vaccinations and COVID-19 infection, and history of menstrual cycle and irregularities, if any.

Sample collection

Saliva samples were collected in the forenoon in a non-stimulating environment. Participants were instructed to abstain from any activities such as drinking and chewing for at least one hour before the procedure. The participants were instructed to clean their mouths with water for a few minutes. Over the course of fifteen minutes, two 1 mL saliva receptacles were filled with samples. The participants were instructed to swallow first, then lunge forward and expectorate it into centrifuge tubes. The whole unstimulated saliva was centrifuged at 1800 rpm and 4°C in a centrifuge for 15 minutes to remove particulates. For biochemical testing, the supernatant was divided into 1 mL aliquots and stored at -80°C. Saliva samples were stored below -20°C until analysis. ELISA was used to measure total antibody (Bio-Rad Platelia SARS-CoV 2 Total AB kit), IgG (Anti-SARS-CoV-2 ELISA IgG kit), and cortisol (DRG Cortisol ELISA 96 T kit) in salivary samples. 

ELISA for detecting total antibody

The procedure was carried out in accordance with the manufacturer's instructions. Significant quantities of saliva (15 µl) were diluted with conjugate (75 µl) (recombinant SARS nucleocapsid protein coupled with horseradish peroxidase) and sample diluent (60 µl) (TRIS-Nacl buffer). To which, 100 µl of the calibrator, positive and negative controls, and samples were transferred to the wells of the reaction microplate and incubated at 37 °C for 30 minutes. The dishes were washed with a microplate washer five times. Equal amounts of the substrate buffer (citric acid and sodium acetate solution) and chromogen (TMB solution) were mixed together, and 200 µl of the resulting solution was put into each well. The plates were then left to sit at room temperature and in the dark for 30 minutes. By adding the stopping solution (1N H2SO4) to each well and vigorously agitating it, the reaction was stopped. Using an ELISA reader (BeneSphera, Avantor, Radnor, USA) with a reference filter of 620 nm, the optical density at 450 nm was measured.

ELISA for detecting IgG immunoglobulin

Before transferring 100 µl of the calibrator, positive and negative controls, and diluted samples to the reaction microplate wells and incubating for 60 minutes at 37°C, the sample was prepared by diluting saliva with buffer at a ratio of 1:1 and mixing once. The plates were washed three times with 300 µl of industrial strength wash buffer. Each well was treated with the enzyme conjugate (100 µl per well of peroxidase-labeled anti-human IgG) and incubated for 30 minutes at 37°C before being rinsed three times. The chromogen/substrate solution was added (100 µl per well) and incubated for 30 minutes at room temperature in the dark followed by the stopping solution. Within 30 minutes of administering the stopping solution, the optical density at 450 nm was measured using an ELISA reader (BeneSphera) with a 620 nm reference filter.

ELISA for detecting cortisol

Equal amounts of standard, control, and sample were added to the wells followed by addition of 200 µl of enzyme conjugate and incubated at room temperature for sixty minutes. Each well was cleansed with 400 µl of diluted wash solution. 100 µl of substrate solution were added and incubated for 15 minutes at ambient temperature. The enzymatic reaction was stopped by adding 100 µl of stop solution to each well. Using an ELISA reader (BeneSphera) with a reference filter of 620 nm, the optical density at 450 nm was measured.

Statistical analysis

To compare the parameters between women with normal menstrual cycles and women who had menopause, the Mann-Whitney test and Kruskal-Wallis Test were used. Spearman’s rho correlation test was used to determine the relationship between cortisol, IgG, and total antibodies. The P-value less than 0.05 was considered as statistically significant.

## Results

A total of 88 participants, 39 of whom were in the normal menstrual phase and 49 of whom had attained menopause, provided saliva samples. Normal menstruating women ranged in age from 21 to 39, while postmenopausal women ranged in age from 40 to 79. Only four participants were not vaccinated, while 15 participants received their first dose. Three participants had a history of COVID-19 infection; two remained positive after the first dose, and one was positive after two doses within two to three months of vaccination. However, they all recovered at home through quarantine and medication.

Saliva samples were tested for three things using ELISA: IgG, total antibody, and cortisol. The best readings were found at 430 nm. This nonparametric study determines normality using the Kolmogorov-Smirnov test. Spearman's rho is utilized to determine the relationship between cortisol, IgG, and total antibody titers. The Kruskal-Wallis test compares more than two groups, whereas the Mann-Whitney test compares only two: women with a normal menstrual cycle and women who have reached menopause.

**Figure 1:**
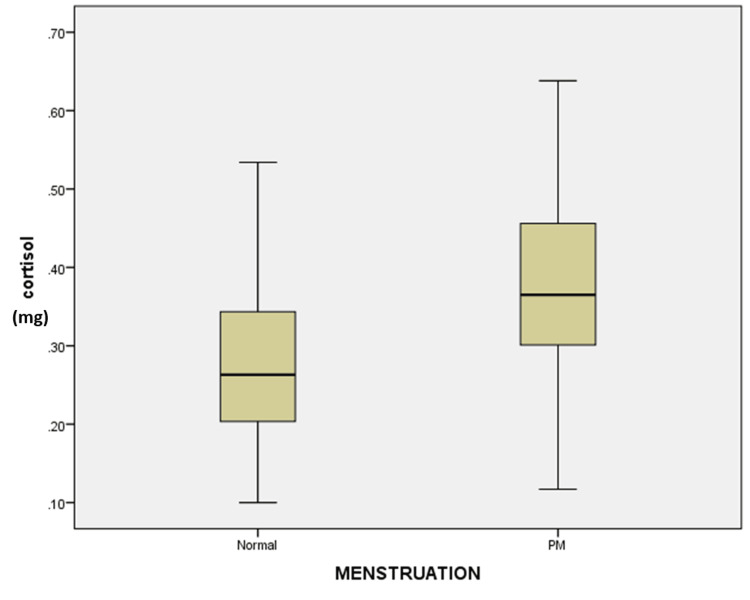
Box diagram displays cortisol levels (mg = milligram) in normal menstruating and postmenopausal (PM) women in the Y-axis & X-axis, respectively The median cortisol levels of women with a normal menstrual cycle are lower than those of women who have reached menopause. The difference was statistically significant (Kruskal Wallis P-value 0.00).

COVID-19-infected women have more IgG antibodies (8.1 mg) than women who have not been infected (5.0 mg). Those who received two vaccinations had higher levels of IgG antibodies (5.6 mg) than those who received only one vaccination or none. Compared to women with a normal menstrual cycle (0.26 mg), postmenopausal women had substantially higher cortisol levels (0.37 mg) (Figure 1).

**Figure 2:**
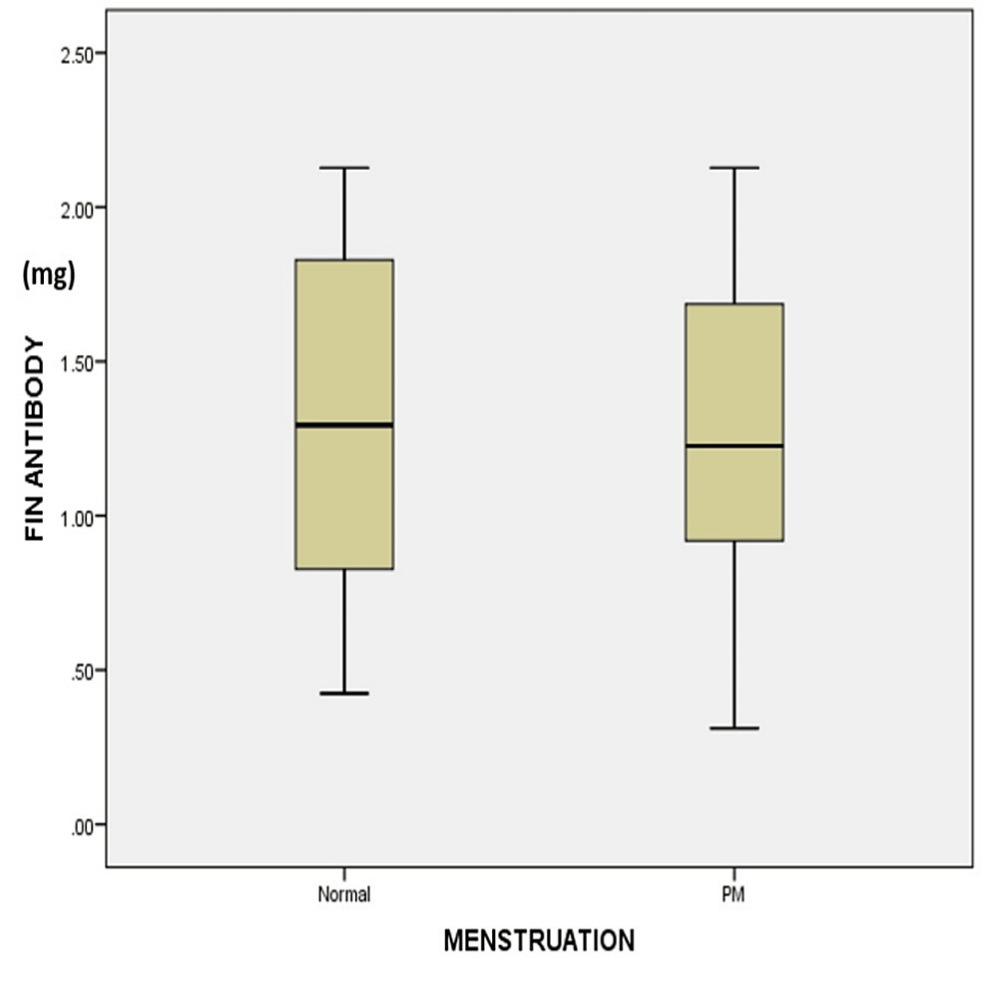
Box plot shows the total antibody (FIN) values (mg = milligram) in normal menstrual and postmenopausal (PM) females in the Y-axis and X-axis, respectively The median total antibody levels of women with a normal menstrual cycle are higher than those of women who have reached menopause. The difference was not statistically significant (Kruskal Wallis P-value 0.76).

The difference was statistically significant (P-value 0.00). Total antibody levels in postmenopausal women were lower than in women with normal menstrual cycles, but the difference is not statistically significant (P = 0.76) (Figure 2).

The difference between the IgG levels of postmenopausal women (8.19 mg) and women with normal menstrual cycles (1.09 mg) is statistically significant (spearman rho P-value = 0.00) (Table 1).

**Table 1:** Spearman's rho correlation between cortisol, total antibody, and IgG The correlation between elevated cortisol levels and elevated IgG antibody levels is statistically significant (Spearman rho P-value = 0.00).

			Cortisol	Total Antibody	IgG
Spearman's rho	Cortisol	Correlation Coefficient	1.000	.421^**^	.261^*^
		Sig. (2-tailed)	.	.000	.014
		N	88	88	88
	FIN Antibody	Correlation Coefficient	.421^**^	1.000	-.046
		Sig. (2-tailed)	.000	.	.669
		N	88	88	88
	FIN IgG	Correlation Coefficient	.261^*^	-.046	1.000
		Sig. (2-tailed)	.014	.669	.
		N	88	88	88
**. Correlation is significant at the 0.01 level (2-tailed).					
*. Correlation is significant at the 0.05 level (2-tailed).					

**Figure 3:**
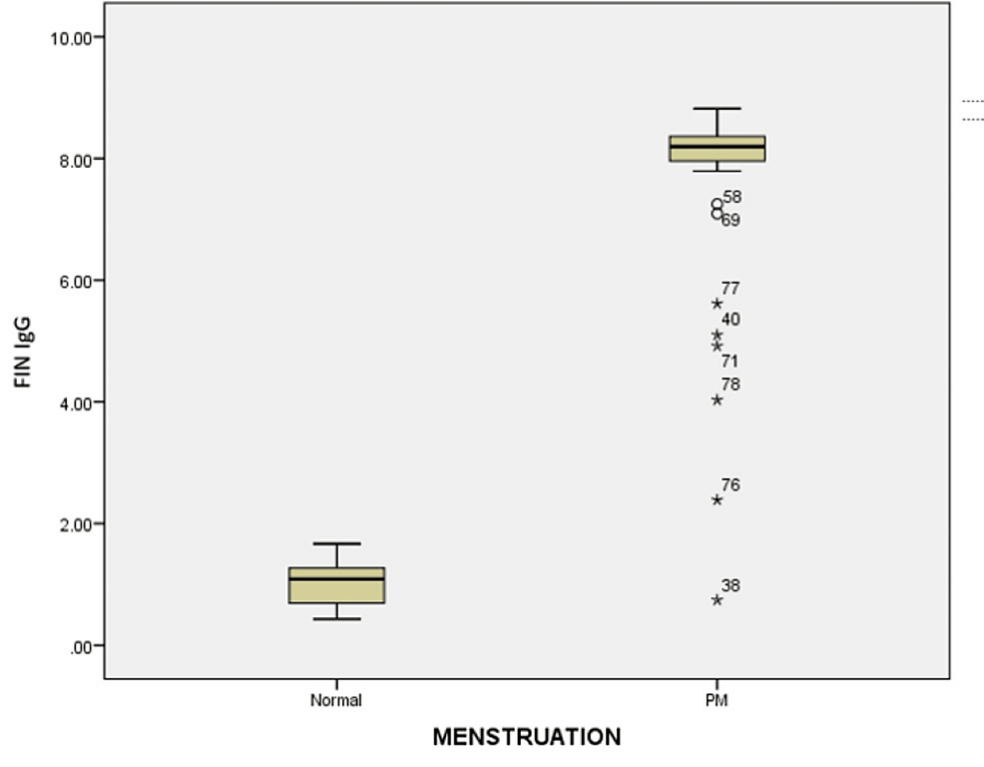
Box diagram displays IgG antibody (FIN IgG) levels (mg = milligram) in normal menstruating and postmenopausal (PM) women in the Y-axis & X-axis, respectively The median IgG antibody levels of women with a normal menstrual cycle were lower than those of women who have reached menopause. The difference was statistically significant (Kruskal Wallis P-value 0.00).

The difference between the IgG levels of postmenopausal women (8.19 mg) and women with normal menstrual cycles (1.09 mg) is statistically significant (spearman rho P-value = 0.00) (Figure 3). 

## Discussion

In a number of crucial crises throughout human history, such as the Ebola and SARS outbreaks and the COVID-19 epidemic that followed, gender has played a significant role [13]. Gender-specific research or investigation on antibodies after vaccination is typically disregarded, despite the fact that this is a cause for concern [14,15]. Few studies have examined the impact of COVID-19 on the female reproductive system. There are few studies on the effects of COVID-19 infection on the female reproductive system. COVID-19 infection has long-term effects on numerous physiological systems, but there are few studies on its effects on the female reproductive system. In order to examine the effects of the COVID-19 vaccination on females, saliva samples were collected.

Cortisol is a steroid hormone produced in the zona fasciculata layer of the adrenal cortex. It regulates the stress response, metabolism, inflammatory response, and immune function [16]. Stress causes an increase in cortisol levels, which results in adaptive alterations in the metabolism, cardiovascular function, and regulation of the immune system. Due to the similarity between the amino acid sequences of ACTH and SARS-CoV-2, the antibodies render endogenous ACTH inactive and eliminate ACTH-secreting cells. A previous study discovered a virus in the adrenal gland as well as necrosis of adrenal cortical cells in COVID-19 patients [17]. Patients who tested positive for COVID-19 have a higher cortisol level than those who tested negative, according to previous research. High cortisol levels are considered a measure of disease severity because they increase mortality [18]. In addition to affecting a person's physical health, COVID-19 also affects their mental health. Due to the fear of illness, isolation, contamination, and loneliness, COVID-19 has had a negative impact on the general population, resulting in psychological crises such as stress, anxiety, and depression. Numerous studies have demonstrated the pandemic impact of fear and anxiety [19,20]. Due to hyperestrogenemia, cortisol levels in women rise during premenopause and menopause. In our study, menopausal women had higher cortisol levels than women with normal menstruation, indicating that the COVID-19 vaccination has no effect on the production of cortisol.

IgA is the predominant immunoglobulin in the buccal mucosa, followed by IgG. IgG antibodies are detectable in the saliva 2-3 weeks after disease onset and persist for nine months, whereas IgA antibodies are detectable one week after disease onset and disappear 4-5 weeks later. IgG antibodies are preferred over IgA and IgM antibodies because their levels remain elevated after infection for a prolonged period of time. After 50 to 60 days after the commencement of symptoms, IgG antibody responses decline [21,22]. Epithelial cells of the salivary glands and buccal mucosa were enriched with COVID-19 entry points, including ACE2 and TMPRSS members, which are believed to be the site of replication and stimulate the production of mucosal antibodies against infection [23]. Infected organs and mucosa express ACE2 and TMPRSS, whereas IgG antibodies to SARS-CoV-2 persist in the saliva of asymptomatic individuals. Antibody concentrations in saliva can be 100-1,000 times lower than serum concentrations. COVID-19 autopsy tissues from the oral cavity exhibited higher viral loads in the minor salivary glands and mucosa during droplet digital polymerase chain reaction analysis of SARS-CoV-2 transcripts. In our study, patients who had recovered from COVID had higher IgG levels than healthy individuals. Moreover, postmenopausal women had more IgG antibodies than women with a normal menstrual cycle. In contrast, normal menstruating women have higher total antibody levels than postmenopausal women. Numerous studies demonstrate that estrogen facilitates the glycosylation of IgG; consequently, postmenopausal women have higher IgG levels, which is consistent with our findings [24]. In addition, IgG levels have increased following the second vaccination dose. Vaccination against COVID-19 may have therefore contributed to the development of humoral immunity in females.

Menopause is a biopsychosocial phenomenon characterized by psychological symptoms such as anxiety, melancholy, illness phobia, hypersensitivity, and irritability [25]. Approximately 30,000 individuals who received COVID vaccination, particularly adenovirus vectored COVID-19 vaccines such as Sputnik V and Johnson & Johnson, reported changes to their menstrual cycles and unanticipated vaginal hemorrhage [26]. Despite vaccination against human papillomavirus, menstrual alterations have been observed [27]. Seventy-five percent of menstruating women infected with SARS-CoV-2 appeared normal, whereas 5% experienced menstrual disruption with increased volume [28]. Comparing lactating and pregnant women to women who are not lactating, the mRNA vaccine induces a comparable level of humoral response in lactating and pregnant women [29]. The immunological influence on female hormones or immune cells in the uterus during the menstrual cycle or the activation of immune cells in the uterine lining may be the cause of immune stimulation in menstruating women, which explains why the immune response among menstruating women following vaccination is greater than in the menopausal group [30]. No patient in our study who received the COVID-19 vaccination reported menstrual irregularities. The gender disparities' causes were not made apparent in the literature. Because it aids in the glycosylation of IgG, we emphasize that it might be caused by female hormones.

The research question was whether saliva can be used as a tool for the detection of COVID-19 as a non-invasive procedure. While RT-qPCR has made significant progress in saliva diagnostics recently, we used ELISA to measure cortisol and antibody levels in female COVID-19 vaccine recipients. The salivary analysis needs to be validated with a large number of samples with multiple parameters. A large-scale follow-up of the study with large samples will be administered in the future.

## Conclusions

We conclude that saliva can be used in preliminary sampling to diagnose and monitor COVID-19. Over time, COVID-19 variants continue to evolve and propagate. This study focuses on the measurement of cortisol and antibodies, as well as the long-term effects of the COVID-19 vaccination in females. After vaccination, female hormones protect against COVID-19 infection via cortisol regulation and IgG antibodies. This is a continuing epidemic that necessitates periodic monitoring of affected individuals to prevent significant systemic damage.
